# Controlling COVID-19 Pandemic: A Mass Screening Experience in Saudi Arabia

**DOI:** 10.3389/fpubh.2020.606385

**Published:** 2021-01-18

**Authors:** Anas A. Khan, Hadil M. Alahdal, Reem M. Alotaibi, Hana S. Sonbol, Rana H. Almaghrabi, Yousef M. Alsofayan, Saqer M. Althunayyan, Faisal A. Alsaif, Sami S. Almudarra, Khaled I. Alabdulkareem, Abdullah M. Assiri, Hani A. Jokhdar

**Affiliations:** ^1^Department of Emergency Medicine, College of Medicine, King Saud University, Riyadh, Saudi Arabia; ^2^Global Center for Mass Gatherings Medicine, Ministry of Health, Riyadh, Saudi Arabia; ^3^Department of Biology, College of Science, Princess Nourah bint Abdulrahman University, Riyadh, Saudi Arabia; ^4^Faculty of Computing and Information Technology, King Abdulaziz University, Jeddah, Saudi Arabia; ^5^Department of Pediatrics, Prince Sultan Military Medical City, Riyadh, Saudi Arabia; ^6^Department of Accident and Trauma, Prince Sultan Bin Abdulaziz College for Emergency Medical Services, King Saud University, Riyadh, Saudi Arabia; ^7^Department of Surgery, College of Medicine, King Saud University, Riyadh, Saudi Arabia; ^8^Field Epidemiology Training Program, Ministry of Health, Riyadh, Saudi Arabia; ^9^Al Imam Mohammad Ibn Saud Islamic University, Riyadh, Saudi Arabia; ^10^Ministry of Health, Riyadh, Saudi Arabia; ^11^Deputyship of Public Health, Ministry of Health, Riyadh, Saudi Arabia

**Keywords:** COVID-19, screening, mass testing, pandemic, Saudi Arabia

## Abstract

A highly accelerating number of people around the world have been infected with novel Coronavirus disease 2019 (COVID-19). Mass screening programs were suggested by the World Health Organization (WHO) as an effective precautionary measure to contain the spread of the virus. On 16 April 2020, a COVID-19 mass screening program was initiated in Saudi Arabia in multiple phases. This study aims to analyze the number of detected COVID-19 cases, their demographic data, and regions most affected in the initial two phases of these mass screening programs. A retrospective cross-sectional study was conducted among the high-risk population as part of the COVID-19 mass screening program across all regions in Saudi Arabia during April and May 2020. A Chi-square-test was used to determine the associations between positive cases and various demographic variables. Out of 71,854 screened individuals, 13.50% (*n* = 9701) were COVID-19 positive, of which 83.27% (*n* = 59,835) were males. Among positive cases, in the 30–39 years age group, 6.36% were in the active phase, and 2.19% were in the community phase. Based on our experience, launching mass screening programs is crucial for early case detection, isolation, and pattern recognition for immediate public interventions.

## Introduction

The emergence of the novel Coronavirus disease 2019 (COVID-19), which was first detected in December 2019 in Wuhan, China, led to the infection of an accelerating number of individuals, causing a local epidemic (1). Shortly after, COVID-19 was a global threat and declared a pandemic by the World Health Organization (WHO) on 11 March 2020 (2). Moreover, the virus can also spread through asymptomatic patients, thus increasing the number of infected patients with an estimated basic reproduction number (R0) that ranges from 2.0 to 3.5 (3, 4). Common symptoms displayed by infected patients include fever, cough, and sore throat. COVID-19 can also lead to serious complications, such as acute respiratory distress syndrome (ARDS), mostly seen in patients with associated comorbidities (5, 6). Although the diagnosis of COVID-19 depends on the epidemiological linkage and clinical presentation, the most reliable method for virus detection is by analyzing respiratory discharges through real-time quantitative polymerase chain reaction (RT-qPCR) (7). Other analyses can be used, including nucleic acid detection, computerized tomography (CT) scan, immune identification of IgM/IgG, enzyme-linked immunosorbent assay (ELISA), blood culture, and a reverse transcriptase PCR (RT-PCR) point of care test, however, with less reliability (8).

Infection with COVID-19 spreads rapidly, with an exponentially growing number infected daily. This has prompted governments to introduce radical measures to control the spread of the virus. Different efforts have been made by various countries to mass test their citizens, to detect new cases, and to evaluate potential solutions (9). The WHO has referred to a fair number of extensive test results, which is between 3–12% of the total number of positive cases (10). The number of people screened in each country has varied depending on several factors, including demographic characteristics, resource availability, and the precautionary measures adopted (11).

The first detected case of COVID-19 in Saudi Arabia was reported by the Ministry of Health (MoH) on 2 March 2020. The patient was immediately quarantined, as well as all his traced contacts (12, 13). By the 12th of September 2020, a total of 325,050 positive COVI-19 cases were detected, 301,836 recovered, and 4,240 cases died (14). This had led to a very low case fatality rate of 1.3% in the country compared with the international case fatality rate of 3.2% (15). Saudi Arabia responded to the pandemic rapidly and imposed several measures to reduce the spread of the infection, including enforced partial curfew hours in multiple cities, as well as suspending events, schools, social gatherings, Umrah, mosques prayers, and business. At one point, a general lockdown was enforced (16–18). In addition to these measures, a national campaign of mass screening was initiated. In the first phase of the campaign, both symptomatic and asymptomatic suspected COVID-19 cases were screened with their close contacts (19). This first phase is also known as the active screening phase involved field teams from MoH targeting intensely populated neighborhoods and labor residential buildings in several cities. Although increasing the number of positive cases detected, this phase helped to contain and locate local outbreaks areas (20). Accordingly, escalated measures were enforced to limit the spread of COVID-19 from these heavily infected areas; an intense lockdown was imposed soon after.

Due to the risk of transmitting the disease from asymptomatic individuals, a second mass screening campaign was initiated. This second phase also known as the community screening phase; targeted low-to-intermediate-risk groups based on their epidemiological risk profile. Risk groups were determined with the aid of the electronic application “Mawid” screening tool (21). Professional health care workers (HCWs) then collected the samples of the targeted population through scheduled appointments in primary care centers. Given the success of these first two phases, the third phase of the mass testing campaign involved screening asymptomatic individuals after applying for electronic appointments through specialized drive through (Takkad) centers, serving more than 2 million beneficiaries from its launch by the end of May 2020 until August 2020 and is still ongoing as planned to continue until the pandemic is eradicated (22).

In this study, our goal was to determine the effectiveness of mass screening programs in Saudi Arabia in the two initial phases by analyzing the number of detected COVID-19 cases, their demographic data, and most regions affected.

## Materials and Methods

The present retrospective cross-sectional study was conducted among COVID-19 screened individuals across all regions in Saudi Arabia. Data from the first two phases of the mass screening program, between 16 April 2020 and 19 May 2020 were included. All screened individuals were included, and there were no exclusion criteria.

**Figure 1:**
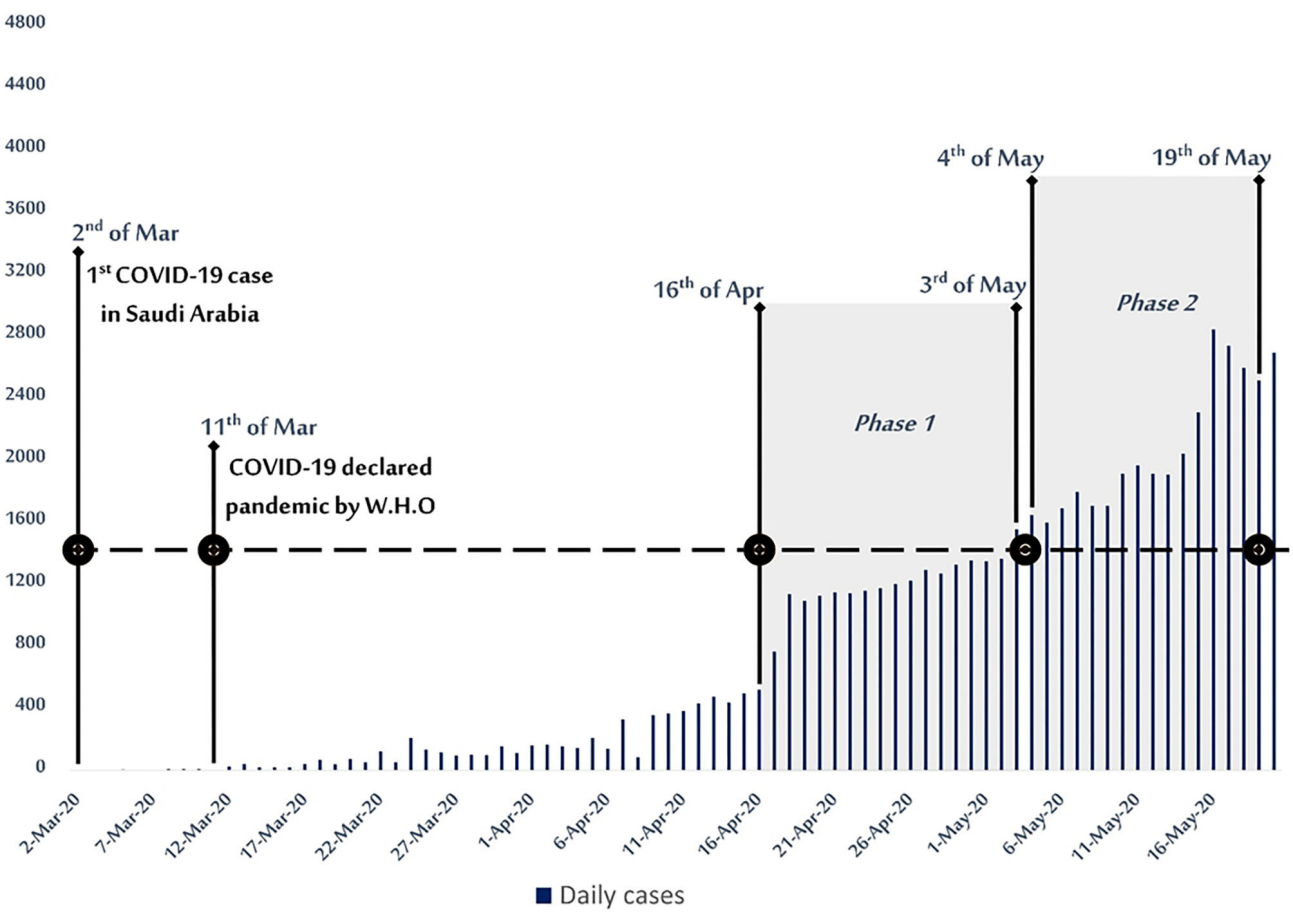
COVID-19 epidemiological curve with active and community mass screening phases between 16 April- 19 May 2020 in Saudi Arabia.

Phase one was defined as an active screening phase performed by the MoH HCWs to screen random individuals in dense districts, between 16 April 2020 and 3 May 2020. Phase two was defined as a community screening phase performed in primary care centers between 4 May 2020 and 19 May 2020. Phase two selected cases based on their epidemiological risk profile through filling self-assessment electronic forms available in the Central Appointment System (Mawid) which is an electronic service originally provided by the MoH to enable patients to book, cancel, or reschedule their appointments at designated primary health care centers. These forms were based on a scoring system with questions about recent travel, contact with confirmed COVID-19 cases, and the presence or absence of specific COVID-19 symptoms. The targeted population in this phase were those with a score of 0-2 (low risk) and 3-4 (intermediate risk) as per MoH screening guidelines (Figure 1) (21, 23).

The confirmatory detecting test in the mass screening was performed by RT-qPCR from Nasopharyngeal swabs following the WHO standardized protocol (24).

The Health Electronic Surveillance Network (HESN) database contains clinical, demographic data, and regions of all screened individuals entered by the HCWs. Positive COVID-19 results, demographic data, and regions of all screened individuals were retrieved from HESN and independently entered by two data collectors into electronic sheets, in which any discrepancies were reviewed and resolved by an assigned investigator. All steps were taken to safeguard data confidentiality and privacy. Ethical approval was obtained from the Institutional Review Board of the Central Committee of the Ministry of Health, KSA, with approval Letter Number 20-115M.

### Statistical Analysis

The data were imported into the most recent version of R, 3.6.3, on the RStudio (1.2.5033) (25). Data were analyzed using the Chi-square goodness-of-fit test to determine the impact of categorical variables, like Saudis/Non-Saudis, gender, age group, and region on test positivity. Follow-up, pairwise comparisons, and Chi-square testing with Bonferroni correction were made to identify which pairs are different from one another concerning COVID-19 test positivity. In the present study, *p*-value < 0.05 was considered significant.

## Results

### Phase 1 (Active Phase)

**Figure 2:**
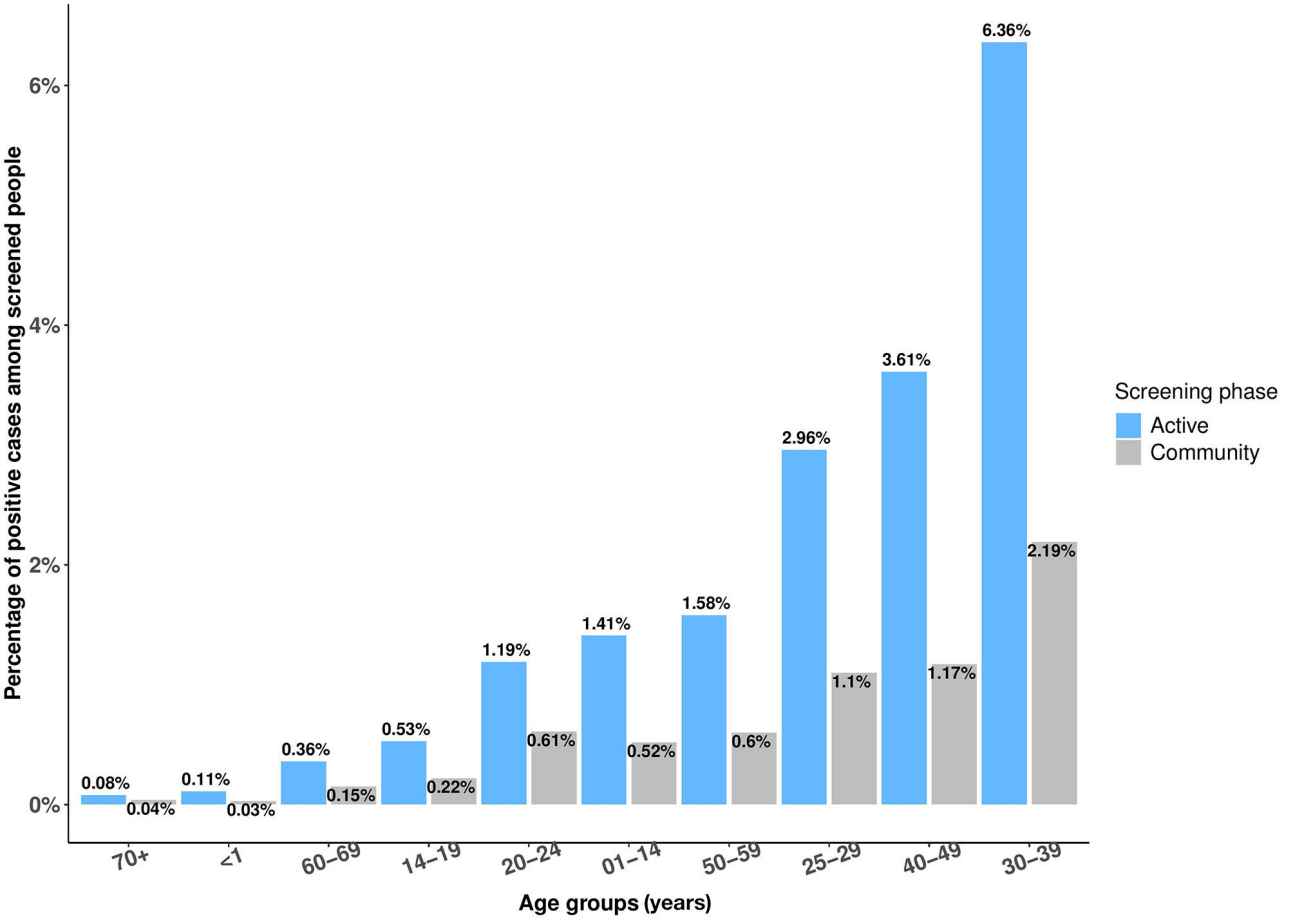
The percentage of positive COVID-19 cases among all screened individuals in Saudi Arabia between 16 April−19 May 2020 in active and community phases in terms of age groups.

In the active phase of mass screening, a total of (42,765) individuals were screened. Positive cases accounted for 18.18% (*n* = 7,776), where 84.13% (*n* = 35,979) of those tested were males (Table 1). Approximately 34.95% (*n* = 15,822) of active phase screened cases were from the aged 30–39 years, representing the highest proportion of positive cases (6.36%, *n* = 2,717), followed by the 40–49 year old and 25–29 year old age groups with 3.61% (*n* = 1,543) and 2.96% (*n* = 1,267), respectively (Figure 2).

**Table 1:** Demographic variables for COVID-19 positive cases during active and community phases for mass screening.

**Phase 1**		**Screened cases *N* = 42,765**	**Positive cases *N* = 7,776 (18.18%)**	***P*-value**
	**Nationality**			=0
	Saudi (%)	11,724 (27.41%)	1,847 (23.75%)	
	Non-Saudi (%)	31,041(72.59%)	5,929 (76.25%)	
	**Gender**			=0.0002
	Male (%)	35,979(84.13%)	6,649 (85.51%)	
	Female (%)	6,786 (15.87%)	1,127 (14.49%)	
	**Age (years)**			=0
	<1	230 (0. 54%)	47 (0.6%)	
	01–14	2,345 (5.48%)	603 (7.75%)	
	14–19	942 (2.2%)	226 (2.91%)	
	20–24	3,541 (8.28%)	509 (6.55%)	
	25–29	7,904 (18.48%)	1,267 (16.29%)	
	30–39	15,822 (36.95%)	2,717 (34.94%)	
	40–49	8,051 (18.83%)	1,543 (19.84%)	
	50–59	3,108 (7.27%)	675 (8.68%)	
	60–69	635 (1.48%)	155 (1.99%)	
	70+	172 (0.4%)	33 (0.42%)	
**Phase 2**		**Screened Cases ***N*** = 29, 089**	**Positive Cases ***N*** = 1,925 (6.62%)**	
	**Nationality**			=0
	Saudi (%)	20,368 (70.02%)	1,018 (52,88%)	
	Non-Saudi (%)	8,721 (29.98%)	907 (47.12%)	
	**Gender**			=0
	Male (%)	23,856 (82.01%)	1,504 (78.13%)	
	Female (%)	5,233 (17.99%)	421 (21.87%)	
	**Age (years)**			=0
	<1	99 (0.34%)	9 (0.47%)	
	01–14	1,305 (4.49%)	150 (7.79%)	
	14–19	1,023 (3.52%)	63 (3.27%)	
	20–24	2,954 (10.16%)	176 (9.14%)	
	25–29	5,213 (17.92%)	319 (16.57%)	
	30–39	11,178 (38.43%)	638 (33.14%)	
	40–49	4,807 (16.53%)	339 (17.61%)	
	50–59	1,803 (6.20%)	174 (9.04%)	
	60–69	524 (1.8%)	43 (2.23%)	
	70+	177 (0.61%)	13 (0.68%)	

**Figure 3:**
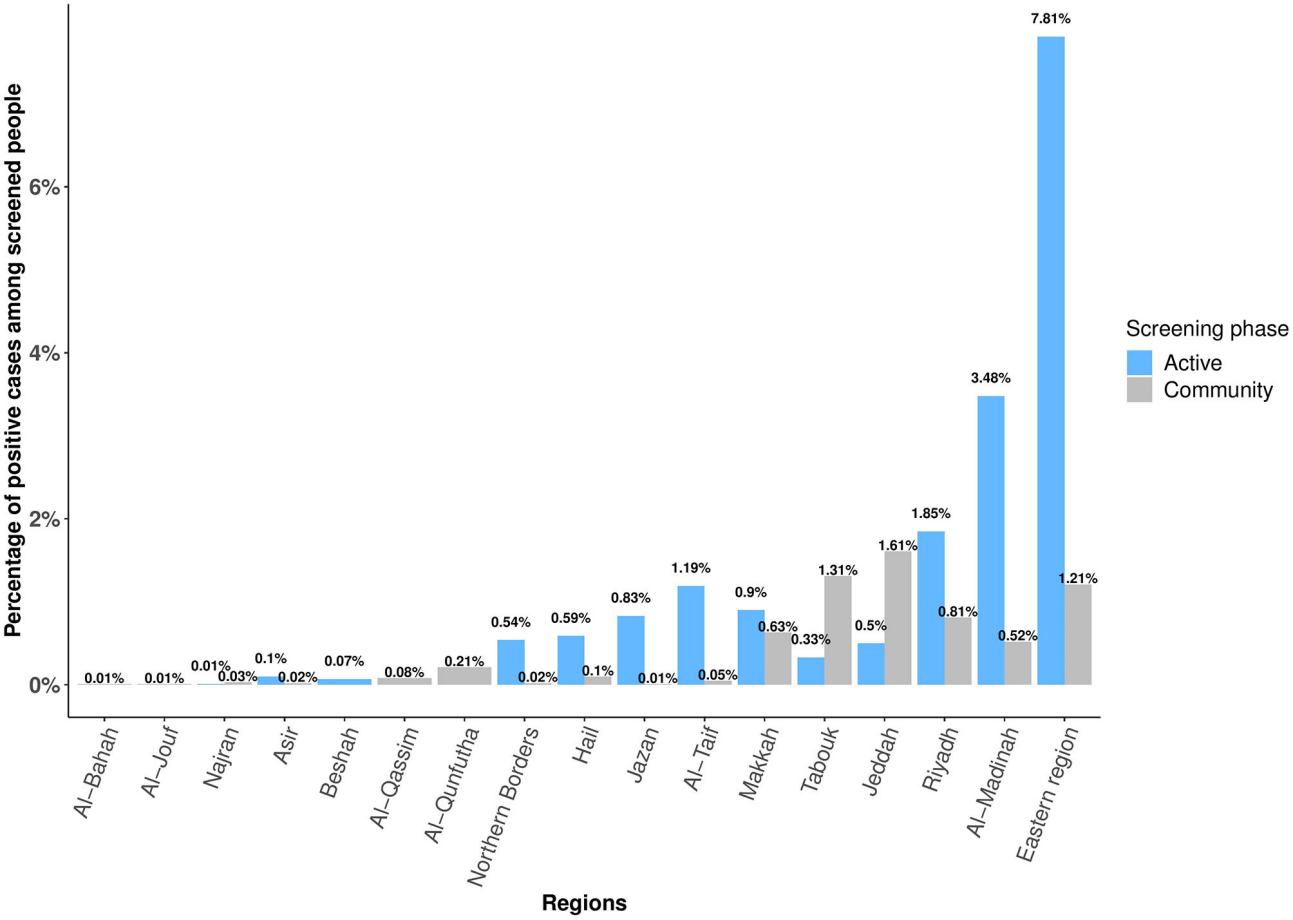
The percentage of positive COVID-19 cases in different regions in Saudi Arabia between 16 April and 19 May 2020 among active and community phases of mass screenings.

The regions with the most positive cases among the active phase screened were the Eastern region, Al-Madinah, and Riyadh, with 7.81% (*n* = 3,339), 3.48% (*n* = 1,490), and 1.85% (*n* = 791), respectively (Figure 3).

Our Chi-square test shows a significant association between the number of positive COVID-19 tests and Saudis/non-Saudis (*p* = 0.0). It also shows a significant correlation between males and females with test positivity (*p* < 0.05). Moreover, different age groups showed significant correlation with positive COVID-19 tests (*p* = 0) (Table 1). For regions and age groups with the number of positive COVID-19 cases, the Chi-square shows a significant association between regions, age groups, and the number of positive COVID-19 test cases (*p* = 0.0002) (Figures 2, 3).

### Phase 2 (Community Phase)

In the community phase screening, a total of (29,089) individuals were screened, of which positive cases accounted for 6.62% (*n* = 1,925) (Table 1). Males accounted for 82.01% (*n* = 23,856) of those screened, of which 78.13% (*n* = 1,504) were positive (Table 1). The most positive cases among the community phase screened individuals were in the 30–39 years old group (2.19%, *n* = 638), followed by the 40–49 years old and 25–29 years old groups: 1.17%, (*n* = 339) and 1.1% (*n* = 319), respectively (Figure 2).

The regions with the most positive cases were Jeddah, Tabouk, and the Eastern region, with 1.61% (*n* = 496), 1.31% (*n* = 381), and 1.21% (*n* = 352), respectively (Figure 3). The Chi-square-test shows a significant association between the number of COVID-19 test-positive cases and Saudi/non-Saudi, gender, age groups, and regions with a *p*-value = 0 (Table 1 and Figures 2, 3).

## Discussion

The COVID-19 pandemic condition continues to devastate most countries around the world. The WHO warned that the pandemic is far from over, and it recognizes the risk of COVID-19 spreading between and within countries and regions. Thus, strategies for detecting and responding to COVID-19 and resource allocation will vary according to national risk assessments. It is recommended by WHO that all suspected cases are tested according to the organization's case definition (24).

Mass screening strategies were adopted by several countries around the world to contain the spread of the virus and to ease lockdown measures. Some of these countries performed massive mass testing programs in which they screened a large number of people daily. Others were moderate in doing mass testing, while others manage to do very few or did not pursue mass testing at all. A model to control the infection was submitted by a group of scientists in the UK, which involved mass screening for the whole population (26). However, this model is difficult to apply due to the massive labor and costs entailed. According to the Economic Cooperation and Development (OECD) report on 30 April 2020, Iceland is considered one of the countries that managed to test a high proportion of its population: one in eight of its population has been tested (9). Like Saudi Arabia, Iceland performed its mass screening in phases; the first mass screening program was followed by another testing campaign to reach about 12% of the population, with a success rate of 93% (9). A high percentage was also achieved in Saudi Arabia; with 45,000 tests been performed daily between May and June 2020, which was then accelerated to 70,000 daily tests in July 2020 (27, 28).

Countries such as Luxembourg and Estonia have achieved a higher rate of mass screening as well. However, these countries are considered sparsely populated nations, which might have helped them to increase the capacity of their mass screening programs (29). Mass screening in highly populated countries can be challenging, even in countries with robust health care systems such as Japan and the UK. However, other highly populated countries have managed to act with great professionalism and efficiency to screen more, such as South Korea and Singapore. South Korea initiated a mass screening program at the end of February 2020, with about 20,000 tested daily. These tests were conducted via mobile examination, drive-through testing, and walk-through testing (30). Singapore managed to test confirmed cases more than once and imposed a whole-nation mass testing campaign, like Saudi Arabia (31). New York state was massively infected with COVID-19, where it adopted precautionary measures that included mass screening tests. A large number were tested daily, which unexpectedly resulted in increasing anxiety among the public. Many were flooding into hospitals and testing centers and queued to be screened, which accelerated the spread of infection (32). Also, the north part of Italy, the crowded tourist region, was heavily infected with the virus. In the early days of the epidemic, the government planned to screen only symptomatic patients. Later on, when the infection had spread to the rest of the country, and the north had contained the spread of COVID-19, they applied population-wide testing for both symptomatic and asymptomatic persons (33, 34). On the other hand, Saudi Arabia has adopted a different approach, as the targeted population in the initial phase of mass screening included both symptomatic and asymptomatic cases which helped extensively to allocate heavily infected areas, thus appropriate measures were applied accordingly with to reduce the infection rates.

Saudi Arabia has carried out a series of ongoing precautionary measures to control the spread of COVID-19 infection and to provide early detection strategies of the disease. When the mass screening program was initiated, the number of infected cases had already exceeded 11,000 cases. The number of cases accelerated after the start of the mass screening program, indicating that positive cases were better detected and allocated (35). Analysis of the mass screening data in the screening phases showed that positive cases in males were significantly exceeding the number of positive cases in females. Similarly, males were more infected than females in Italy (82%), the USA (61.3%), and China (54.3%) (36–38). This can be explained by several factors including that more men are involved in the workforce than women and that men are more susceptible to be infected with the virus than women (39, 40).

The non-Saudi cases were higher in the active phase compared with the community phase. This is likely because most of the screened people in the active phase were from densely populated districts and worker housing (20). However, the community phase was targeting individuals based on their low to intermediate epidemiological risk profile after filling electronic screening tools. Thus, as expected, most of the screened were Saudi (60.7%) (21, 41).

In terms of age, our data showed that the 30–39 years old group was the most screened in both phases, which has also been observed in other countries, such as the USA and China (42–44). It was expected that this age group would be the most infected in both phases, considering that workers and most of the Saudi population are within this age group.

Regarding the overall coverage of the mass screening program across regions in Saudi Arabia, most were screened in both phases. Regions such as Eastern and Riyadh had a larger number of screened individuals compared to Makkah and Al-Madinah, despite that mass screening centers were equally distributed in most Saudi regions. These variabilities can be attributed to several factors, including peoples' lack of awareness, being afraid of the test, as well as being worried about positive test results (45, 46). Thus, it is essential to improve risk communication and community engagement regarding COVID-19 pandemic (47). The 1st reported COVID-19 case entered through the Saudis Eastern region port of entry leading to the lockdown of a number of its Governorate in the early phases of the pandemic (5, 48). This would explain the high positivity rate of cases in the Eastern region in the initial active phase, which was eventually reduced in the community phase because of effective curfew measures. Additionally, a high number of positive cases in phase one in both Al-Madinah and Riyadh regions was observed, which could be attributed to the high number of dense districts easing the spread of the virus. This led to enforce lockdown on these dense areas in the early phases of the pandemic to reduce the number of positive cases in Al- Madinah and Riyadh (49, 50). Despite more people were being aware about screening services at designated primary care centers in phase two, nevertheless Jeddah and Tabouk regions were the highest in the number of positive cases, which can be explained by unstable epidemiological situation in both regions and lower level of awareness about precautionary measures. This increase in the number of cases led to impose further curfew measures to curb the spread of the disease (14, 49, 51).

Based on the COVID-19 mass screening experience in Saudi Arabia, 13.50% of all screened individuals (71,848) in the initial two phases were positive. This percentage nearly falls in the recommended percentage of an adequate number of tests as suggested by the WHO; 3%-12% of positive cases of the total screened people. Despite the number of tests in these two phases can be considered small, still, these phases helped to locate heavily infected areas and introduced appropriate measures to control the spread of the infection. Due to the finding that most of the positive cases were from densely populated areas and within the 30–39 years old group, it is crucial to focus on increasing the level of community awareness, especially among those targeted populations (47, 52).

To our knowledge, this is one of the initial studies to address mass screening in Saudi Arabia. However, this study has some limitations. First, some variables were missing in the electronic database (HESN), such as clinical characteristics, patients' disposition, and disease outcomes. Further analysis of these variables could have been achieved to describe detailed demographic data of cases screened, map the disease severity, and guide targeted areas for mass screening. Second, the community screening phase included those with a pre-defined epidemiological risk profile, excluding many other cases that could have an added value to our results. Third, the mass screening program will continue, with additional phases, but this study addressed only the first two phases limiting the generalizability of the results in this study.

## Conclusion

Launching Saudi Arabia's mass screening program during the early phases of the pandemic was a helpful epidemiological surveillance tool, which was based on accumulative experiences with previous outbreaks such as MERS CoV. Phase 1 showed high COVID-19 positive cases in densely populated areas, males, and age groups between 30–39 years. Effective awareness campaigns for these groups are critical to contain the infection. A high number of COVID-19 cases in phase 2, in Jeddah, Tabouk, and the Eastern region, was expected due to their unstable epidemiological situation at that specific time interval. Following screening phases of the mass screening program should address gaps from earlier phases which include screening more individuals, using rapid screening techniques, and providing more reliable results in less time to limit the spread of COVID-19.

## Data Availability Statement

The original contributions generated in the study are included in the article/supplementary materials, further inquiries can be directed to the corresponding author.

## Ethics Statement

The studies involving human participants were reviewed and approved by The central IRB-MoH, No: 20-115M, GCMGM-MoH. Written informed consent for participation was not required for this study in accordance with the national legislation and the institutional requirements.

## Author Contributions

AK, HA, RMA, and YA: worked on concepts. HA, YA, HS, and AK: design. HA, RHA, HS, and YA: literature search, writing, and manuscript preparation. SMA, SSA, AA, and HJ: data acquisition. RMA and HA: statistical analysis. HA, AK, FA, and RA: manuscript editing. AK, HJ, AA, and SMA: manuscript review. AK: guarantor. All authors: contributed to the article and approved the submitted version.

## Conflict of Interest

The authors declare that the research was conducted in the absence of any commercial or financial relationships that could be construed as a potential conflict of interest. The reviewer MM declared a shared affiliation with several of the authors YA, SSA, AA, and HJ to the handling editor at time of review.
